# Leveraging quality improvement to promote health equity: standardization of prenatal aspirin recommendations

**DOI:** 10.1186/s12884-023-05922-w

**Published:** 2023-09-08

**Authors:** Maya E. Gross, Amy Godecker, Ainsley Hughes, Katherine Sampene

**Affiliations:** 1 Department of Obstetrics and Gynecology, University of Wisconsin School of Medicine and Public Health, Madison, WI USA; 2 Department of Obstetrics and Gynecology, George Washington School of Medicine, Washington, DC USA

**Keywords:** Preeclampsia, Aspirin, Pregnancy, Quality improvement, Implementation

## Abstract

**Objective:**

Aspirin (ASA) is recommended for patients at elevated risk of preeclampsia. Limited data exists on adherence to guidelines for ASA prescription. This project evaluates the implementation of a standardized approach to ASA prescription in an academic OB/Gyn practice.

**Methods:**

We implemented a quality improvement project to evaluate compliance with the United States Preventative Services Task Force (USPSTF) recommendations for ASA to prevent preeclampsia. Pre-intervention, we analyzed prescription adherence at 201 New Obstetric (NOB) visits. A multi-step intervention was then implemented at 199 NOB visits. Nurses utilized a checklist created from USPSTF guidelines to identify high-risk patients, defined as having ≥1 high-risk factor or ≥2 moderate-risk factors. ASA orders were placed by physicians. A Plan-Do-Study-Act (PDSA) cycle was performed, and changes implemented. Primary outcome was percent of patients screened at RN intake visit (goal = 90%). Secondary outcomes were percent of patients who screened positive that received the ASA recommendation (goal = 80%) and percent screened and recommended by race.

**Results:**

Pre-intervention, 47% of patients met criteria for ASA and 28% received a documented recommendation. Post-intervention, 99% were screened. Half (48%) met criteria for an ASA recommendation and 79% received a recommendation (p = < 0.001). Rates of appropriate recommendation did not differ by Black (80%) vs. non-Black (79%) status (p = 0.25). Subsequent PDSA cycles for 12 months neared 100% RN screening rates. Physicians correctly recommended ASA 80–100% of the time.

**Conclusion:**

It is feasible, sustainable and equitable to standardize screening and implementation of ASA to patients at high risk for preeclampsia. Providers can easily reproduce our processes to improve delivery of equitable and reliable preventative obstetric care.

## Introduction

Hypertensive disorders of pregnancy are common, affecting 10–12% of all pregnancies [1]. Preeclampsia and gestational hypertension (GHTN) affect up to 8% and 3% of pregnancies in the United States, respectively, and the incidence of each rose steadily from 1987-2004 [1–4]. In the United States and other high-income countries, up to 16% of maternal deaths and up to one fourth of all medically indicated preterm births can be attributed to hypertensive disorders [2–4]. Patients who identify as Black are disproportionately affected by both the incidence and implications of preeclampsia, with this group of patients experiencing the highest rates of early-onset preeclampsia and preeclampsia-associated morbidity and mortality [5–13].

**Fig. 1:**
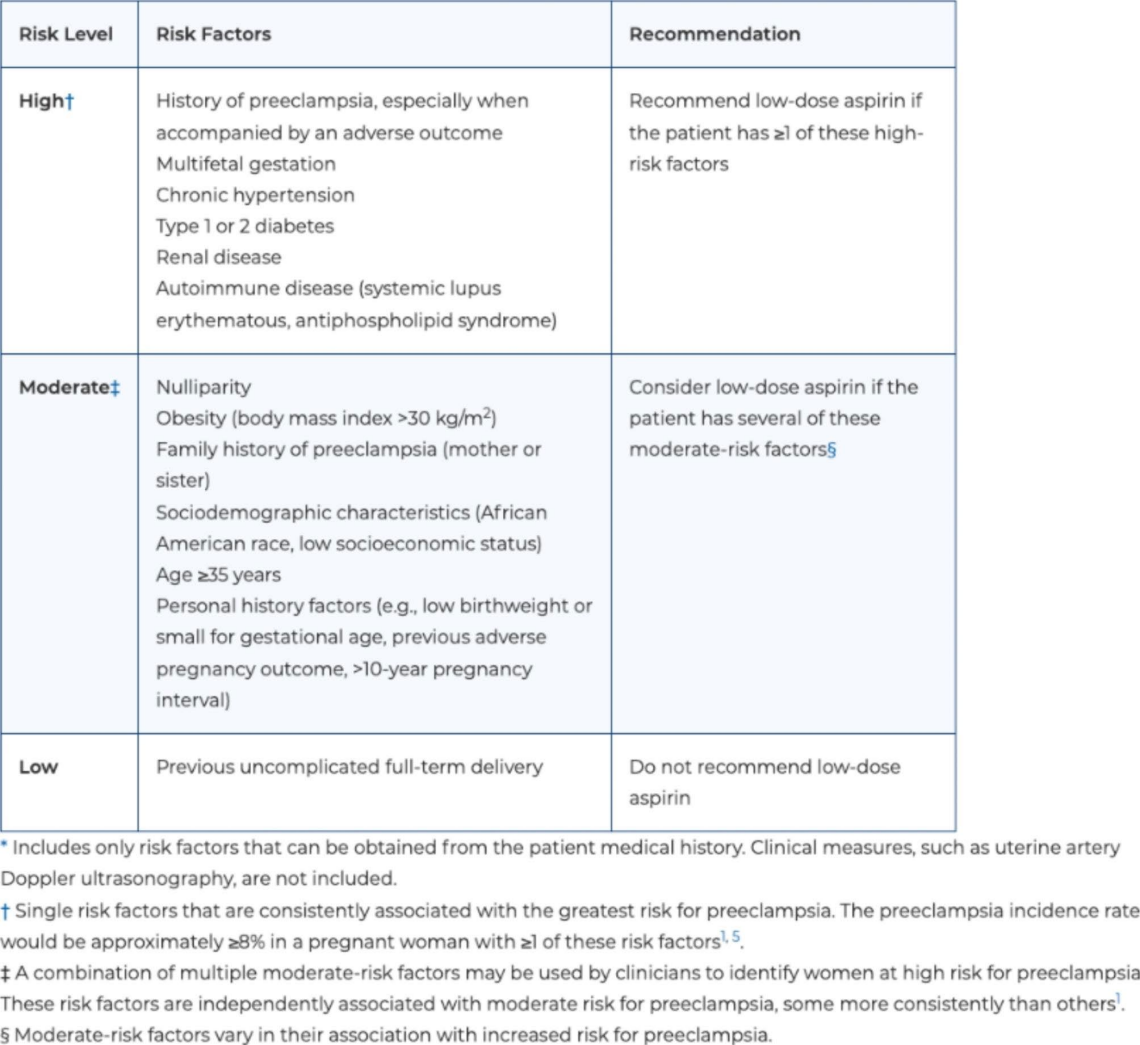
2014 USPSTF Aspirin for Preeclampsia recommendations. Reproduced from: https://www.uspreventiveservicestaskforce.org/uspstf/recommendation/aspirin-to-prevent-cardiovascular-disease-preventive-medication

**Fig. 2:**
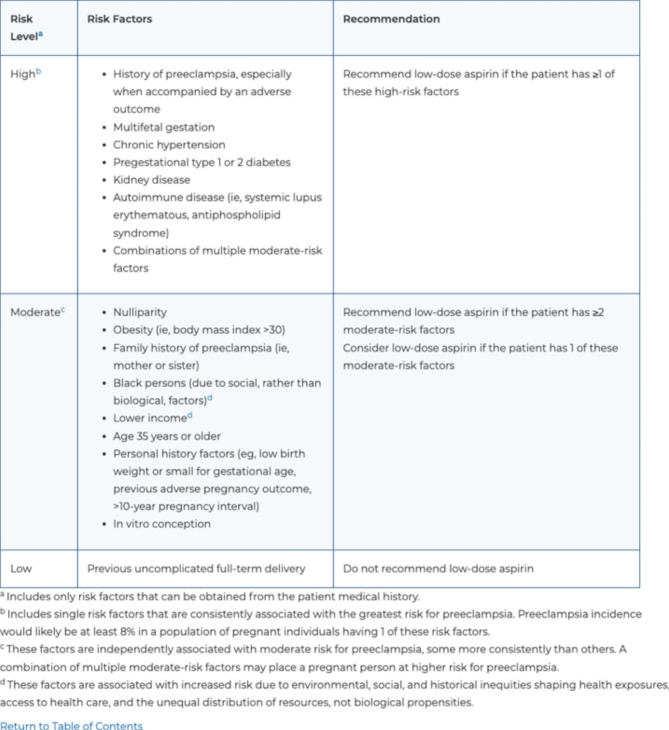
2021 USPSTF Aspirin for Preeclampsia recommendations. Reproduced from: https://www.uspreventiveservicestaskforce.org/uspstf/recommendation/aspirin-to-prevent-cardiovascular-disease-preventive-medication

Preeclampsia has been identified as one of the most preventable causes of maternal morbidity and mortality [5, 14]. Daily aspirin (ASA) in pregnancy has been recommended for patients at high risk of developing preeclampsia since November of 2013, when the Hypertension in Pregnancy Task Force Report was issued by the American College of Obstetricians and Gynecologists (ACOG) [13, 15]. ASA can reduce the risk for preeclampsia by 15–62%, with maximum benefit when started prior to 16 weeks gestation [3, 16]. ASA has also been demonstrated to significantly reduce the risk of fetal growth restriction, perinatal mortality, and preterm birth [3, 15, 16]. The recommendation to prescribe ASA to women at high risk of preeclampsia was taken up, and expanded upon, by the U.S. Preventive Services Task Force (USPSTF) in 2014 and reaffirmed in 2021 (Figs. 1 and 2) [16, 17].

Various methods exist to screen for patients at high risk of developing a hypertensive disorder of pregnancy, including personal and family history, ultrasonography, and serum biomarkers [3, 15, 18, 19]. While the use of serum biomarkers and ultrasonography can provide useful information in addition to clinical and family history, their use is not always available in clinical practice and recommendations for use are inconsistent [3, 15, 18]. To capture this at-risk population, the USPSTF developed a history-based screening algorithm consisting of several high and moderate risk factors (Figs. 1 and 2) [16]. All pregnant patients with at least one high-risk factor or at least two moderate-risk factors are recommended to be prescribed daily low dose ASA. Ideally, ASA should be initiated before 16 weeks of gestation and continued daily throughout the pregnancy, but can be prescribed at any point between 12 and 28 weeks gestation [16].

Data on provider adherence to USPSTF guidelines and practice patterns surrounding prescription of ASA to pregnant patients at moderate or high risk of developing preeclampsia are limited, but available evidence suggests poor compliance [20]. Standardization is one method which has been proven in multiple industries to create safer work environments, decrease bias and improve outcomes [21–24]. The Institute of Medicine urges health care systems to incorporate established principles including standardization in order to improve patient safety, and ACOG echoes this call [23, 25]. Standardization has been successfully implemented in obstetrics and gynecology in both inpatient and outpatient settings, including standardization of cesarean delivery technique and group B streptococci screening [25, 26]. Standardization has been documented to decrease disparities by reducing the effect of implicit bias, to improve efficiency and decrease rates of avoidable complications [24–26]. Quality improvement, specifically equity-focused quality improvement, has been posited as a tool for the reduction of health disparities [27].

We aimed to improve understanding of and adherence to established guidelines recommending prescription of ASA to pregnant patients at moderate to high risk of developing preeclampsia at our institution. We also aimed to assess whether standardization of ASA (ASA) screening and prescription resulted in equitable distribution of indicated ASA recommendations for patients who self-identified as Black, versus non-Black. Our goal was to accurately recommend ASA to at least 80% of all patients warranting the recommendation.

## Methods

We performed a two-part quality improvement initiative within our institution’s general obstetrics and gynecology group, which includes 3 clinic sites in an academic generalist OB/Gyn group of 23 physicians and 8 advanced practice providers at the University of Wisconsin Hospitals and Clinics. This project was deemed not human subjects research by the University of Wisconsin Institutional Review Board as it is a quality improvement initiative. Part 1 consisted of a pre- and post-intervention analysis of 200 new obstetric (NOB) visits in our practice. Part 2 utilized the Model for Improvement to conduct monthly Plan-Do-Study-Act (PDSA) cycles, analyzing preeclampsia screening and ASA prescription practices on a monthly basis over a period of 12 months during implementation of our intervention [28]. Each month, charts were randomly selected and analyzed until 10 patients were identified that should have received an ASA recommendation.

Patients were included if they presented to our clinics prior to 16 weeks of gestation for a NOB visit. Patients were excluded from analysis if they had a transfer of care into the practice after 16 weeks, if delivery occurred before 20 weeks of gestation or if their pregnancy ended in a miscarriage or intrauterine fetal demise (IUFD) that was not related to a hypertensive disorder of pregnancy.

We collected baseline rates of recommendation for ASA in all NOB patients across all clinics within our practice for one month in November 2019 for our pre-intervention data set. November 2019 was chosen as a pre-intervention timeline as we wanted to reflect “typical” practice prior to the beginning of the COVID-19 pandemic in early 2020. We launched our quality improvement initiative in January 2021 and continued surveillance of screening and ASA recommendations through December 2021. Following three months of our intervention, we performed a post-intervention chart review of one month of additional NOB visits, with an additional category of rate of screening to determine eligibility for ASA. Our 199 post-intervention charts were collected from March-April 2021.

**Fig. 3:**
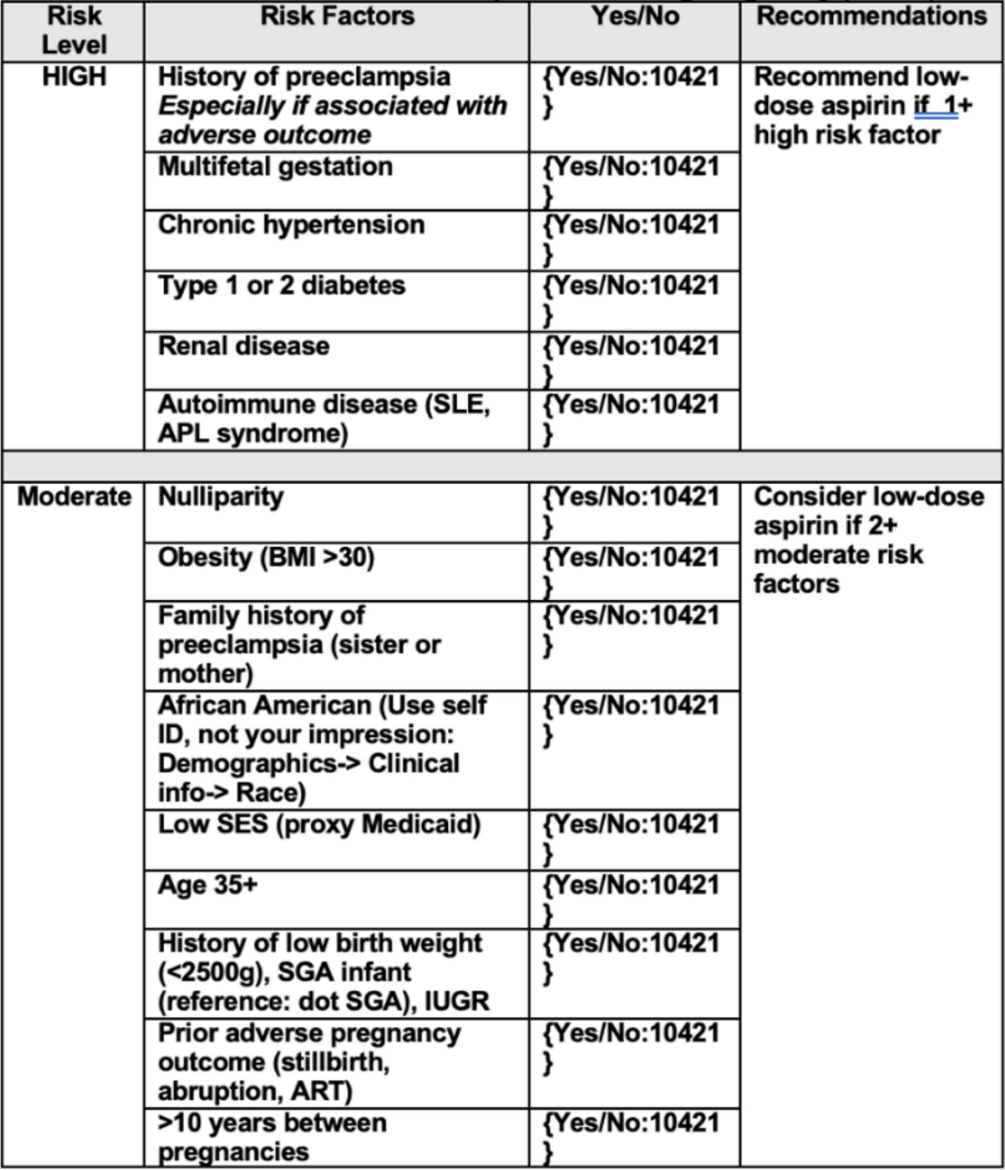
RN Screening Workflow

Detailed review of each chart was performed by two members of the project team to identify moderate- and high-risk factors for the development of preeclampsia, based on the USPSTF 2014 ASA screening guidelines, which were the most updated version available at time of data collection (Figs. 1 and 3). We made an intentional decision to add GHTN to the “high-risk” criteria, as this is generally considered be commensurate with the 8% or higher risk of preeclampsia, as outlined by the USPSTF for warranting an ASA recommendation. Data was stored in an encrypted department drive which is compatible with Health Insurance Portability and Accountability Act (HIPAA.) We analyzed clinic notes for documentation regarding discussion of recommendation for ASA or for orders for ASA placed in the chart during the pregnancy.

Data analysis of “correctness” of recommendation was completed based on the 2021 guidelines. Our clinic practice was to recommend ASA for patients with two moderate risk factors for ASA, based on the “consider” recommendation from the 2014 guidelines. Given the update to “recommend” in the 2021 guidelines, our practice aligned with updated USPSTF recommendations.

The intervention consisted of a nursing-performed screening using the USPSTF Clinic Risk Assessment, collected during scheduled NOB RN telephone visits, which are conducted with all newly pregnant patients joining the practice. We developed a workflow after meeting with provider and nursing leadership to determine feasibility and acceptability among nursing staff. The workflow was created and embedded into the existing telephone note utilized by nursing staff (Fig. 3).

Nurses would document number of high and moderate risk factors, whether the patient met criteria for an ASA recommendation (with ≥ 1 high or ≥ 2 moderate risk factors.) For patients meeting criteria for ASA, nurses would pend an order for 81 mg ASA in the NOB provider encounter [15]. During the in-person new OB visit with the provider, providers would review the screening performed by the RN to confirm need for ASA recommendation, discuss the recommendation with the patient, and sign the order.

The primary outcome was the percent of patients screened with a goal of 90% screening rate. The secondary outcomes included the percent of patients who screened positive that received the ASA recommendation with a goal of 80%, percent of patients who screened negative who were incorrectly recommended ASA, with ongoing screening and recommendation compliance analyzed over the subsequent 12 months. We analyzed the primary and secondary outcomes by whether the patient self-identified as Black (alone or in combination with another race), as recorded in the electronic health record. This stratification of self-identified race was in concordance with the USPSTF and ACOG guidelines for risk stratification of preeclampsia, in which self-identified Black race is an individual risk factor.

We performed descriptive statistics using frequencies to measure risk factors for preeclampsia in our pre- and post-intervention groups and outcomes in our pre- and post- intervention analysis (Part 1). We used two-sided Fisher’s exact tests to test for significant differences (p < 0.05) in risk factors between the groups, and for outcomes differences between our pre- and post-intervention cohorts. We also performed PDSA cycles with randomly selected NOB charts each month, with in-depth chart review to identify barriers or challenges to appropriate screening and prescription throughout our intervention. In-time adjustments were made monthly based on findings from this detailed chart review. Run charts were utilized to report rates of change over the 12 months of follow-up.

## Results

Pre- and post-intervention cohorts included for analysis consisted of 201 and 199 patients, respectively. Baseline distribution of risk factors for preeclampsia was similar in pre- and post-intervention cohorts (Table 1).

**Table 1:** Preeclampsia Risk Factors for Pre- and Post-Intervention Cohorts

Risk Factors, n (%)	Pre-intervention (n = 201)	Post-intervention (n = 199)	Exact p-value**
High Risk Factors			
History of preeclampsia/GHTN	23 (11.4)	14 (7.0)	0.167
CHTN	4 (2.0)	9 (4.5)	0.171
Multifetal gestation	5 (2.5)	6 (3.0)	0.770
Pregestational diabetes	1 (0.5)	2 (1.0)	0.622
Kidney disease	0 (0.0)	0 (0.0)	1.000
Autoimmune disease	1 (0.5)	1 (0.5)	1.000
Moderate Risk Factors			
Nulliparity	86 (42.8)	76 (38.2)	0.361
Obesity	71 (35.3)	67 (33.7)	0.753
Family history preeclampsia	1 (0.5)	5 (2.5)	0.121
Black race	24 (11.9)	27 (13.6)	0.655
Lower income	32 (15.9)	37 (18.6)	0.510
Age ≥ 35	45 (22.4)	37 (18.6)	0.387
History of low birth weight infant, SGA infant, FGR	8 (4.0)	6 (3.0)	0.787
Other personal history factors	13 (6.5)	9 (4.5)	0.512
>10 years between pregnancies	4 (2.0)	3 (1.5)	1.000

*Other personal history factors include previous adverse pregnancy outcomes, IVF pregnancies

**Two-sided Fisher’s exact test for differences in pre-intervention vs. post-intervention groups

In both cohorts, prior history of GHTN or preeclampsia were the most common high-risk factors identified. Nulliparity, obesity, and maternal age ≥ 35 years were the most common moderate-risk factors identified.

Pre-intervention, 47% of overall patients met criteria for ASA (Table 2).

**Table 2:** Aspirin Screening and Recommendations for Pre- and Post-intervention Cohorts, by Self-identified Black or Non-Black Race

Characteristic or Outcome, n (%)	Pre-intervention (n = 201)	Post-intervention (n = 199)	Exact p-value
Patients			
Black	24 (11.9)	27 (13.6)	0.655*
Non-Black	177 (88.1)	172 (86.4)	0.655*
Screened for aspirin	N/A	197 (99.0)	
Black	N/A	26 (96.3)	0.254**
Non-Black	N/A	171 (99.4)	
Candidates for aspirin	94 (46.8)	95 (47.7)	0.920*
Black	21 (87.5)	25 (92.6)	< 0.001**
Non-Black	73 (41.2)	70 (40.7)	
Correct recommendation for aspirin	26 (27.7)	75 (78.9)	< 0.001*
Black	6 (28.6)	20 (80.0)	1.000**
Non-Black	20 (27.4)	55 (78.6)	
Incorrect recommendation for aspirin	5 (4.7)	11 (10.6)	0.124*
Black	0 (0.0)	0 (0.0)	1.000**
Non-Black	5 (4.8)	11 (10.8)	

*Two-sided Fisher’s exact test for differences in pre-intervention vs. post-intervention groups

** Two-sided Fisher’s exact test for differences in post-intervention Black vs. non-Black groups

Of these patients, 28% received a correct recommendation for ASA, while 72% correctly did not receive a recommendation for ASA. Post-intervention, 99% (n = 197) of patients were screened using our workflow. For the two patients who did not have screening performed, the NOB telephone visit was documented using an alternative (outdated) template which did not include the ASA screening table. Consistent with pre-intervention data, roughly half (48%) of our post-intervention cohort met criteria for ASA. Rates of correct ASA recommendation increased from 28% pre-intervention to 79% post-intervention (exact p-value < 0.001). There was no significant different from pre-intervention to post-intervention in the rate of incorrect recommendation for ASA (5% vs. 11%; exact p-value 0.124).

When results were analyzed by race, patients who self-identified as Black were more than twice as likely to meet criteria for ASA compared to patients not identifying as Black (93% vs. 41%; exact p-value < 0.001) (Table 2). Rates of correct ASA recommendation for patients identifying as Black were similar to the non-Black population in both our pre- and post- intervention cohorts; after the intervention, 80% of Black and 79% non-Black patients received a correct ASA recommendation (exact p-value 1.000). Similarly, there were no pre- or post-intervention differences between Black and non-Black patients receiving incorrect recommendations for ASA (exact p-value 1.000).

**Fig. 4:**
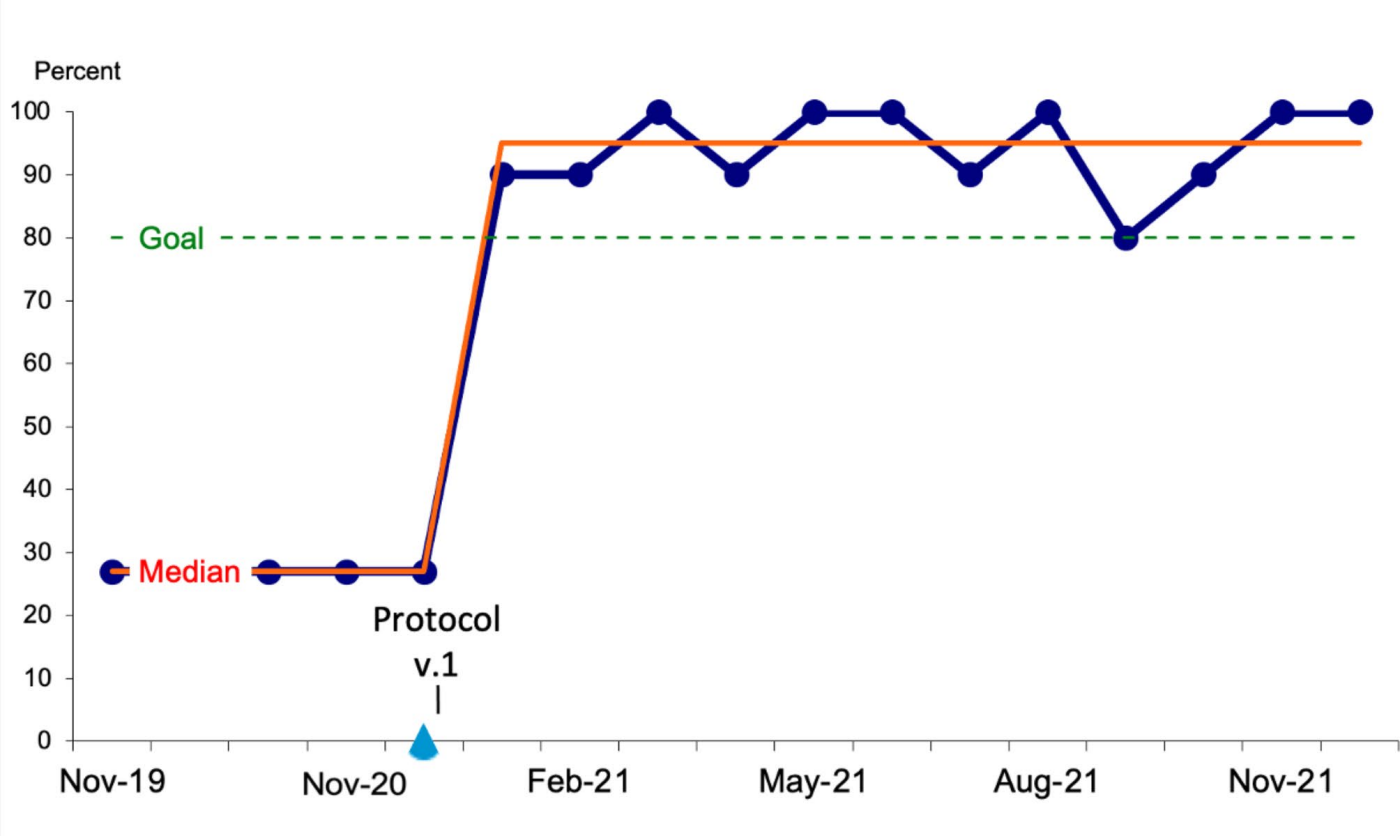
Frequency of correct aspirin recommendations by month, for a duration of 12 months post-intervention. Intervention began in January of 2021

In PDSA cycles performed over a twelve-month period after the initial intervention was implemented, rates of screening were 100%. Correct ASA recommendations were made for 80–100% of patients meeting criteria monthly (Fig. 4).

Patient charts which received incorrect recommendations for or against ASA were reviewed in detail to identify areas for improvement. Lack of clarity surrounding medical terminology was identified as a contributing factor to incorrect screenings in a number of cases; changes in the screening workflow were made to clarify definitions of kidney disease (04/2021), autoimmune disease (06/2021), parity (07/2021), and in vitro conception (07/2021) to address this issue. Additional changes included adjusting the location of documentation in the electronic health record to ensure providers could easily find documented risk factors. During the implementation year, providers were reminded about the workflow and recommended documentation at department-wide meetings (02/2021 and 08/2021) and the workflow was also adjusted to incorporate updates from the 2021 USPSTF guidelines for ASA prescription.

## Discussion

In this quality improvement initiative, rates of indicated ASA recommendations to women at moderate to high risk of developing preeclampsia increased by nearly three-fold after implementation of a standardized screening by intake nurses for NOB patients. This improvement was sustained over a 12-month period of monitoring after the initial implementation.

We designed our quality improvement initiative as a nursing-driven process. Rationale for this system was two-fold. First, incorporating a screening checklist into the existing nurse intake phone call ensures standardized, reliable screening for all new OB visits in the practice, in which obstetric providers operate out of multiple clinic sites. Nurses use a template which undergoes periodic review with practice leadership, minimizing variation between providers, with a goal of reducing error and bias in screening practices. Second, the USPSTF checklist is complicated, with 15 separate informational components obtained from a combination of chart review and history-taking. In an already busy NOB visit, providers are often stretched for time. Data shows that alert and checklist fatigue have a direct correlation with provider burnout and well-being [29, 30]. By allocating the screening process to qualified nursing staff, providers can focus on the discussion surrounding preeclampsia and rationale for prescription.

In our screening workflow, history of GHTN was included as a high-risk factor for development of preeclampsia. This factor is not separately included in the USPSTF screening algorithm [16]. Included criteria for ASA prescription in the USPSTF recommendations stem from an estimated risk of developing preeclampsia of 8% [16]. Patients with a history of GHTN in a prior pregnancy are known to have an increased risk of recurrent hypertensive disease; based on previously reported studies, the authors of this publication believe that history of prior GHTN will often place patients above this cutoff in a subsequent pregnancy [15, 16, 31–35]. Additionally, doubt exists whether GHTN represents a true separate diagnosis from preeclampsia without severe features rather than a different point on the hypertension in pregnancy spectrum, and GHTN progresses to preeclampsia in up to 50% of cases [1, 4, 31–33].

We included self-identified Black race as an independent moderate-risk factor for the development of preeclampsia, in line with recommendations from the USPSTF and ACOG [3, 16, 36]. There are well-documented, persistent racial and ethnic disparities in the incidence and morbidity associated with preeclampsia, with Black patients experiencing the highest rates of morbidity and mortality related to preeclampsia [5–13, 27, 35]. In light of recent, justified, critiques of race-based risk calculators, the inclusion of race as a risk factor or clinical variable guiding clinical decisions should be scrutinized [37]. The Vaginal Birth After Cesarean (VBAC) calculator, which previously included Black race as a negative predictor of successful VBAC, is a timely example of potentially harmful inclusion of race in clinical tools.

The recently published article in the NEJM, Hidden in Plain Sight, provided guidelines when considering inclusion of race in health calculators [37]. First, practitioners should question whether the inclusion of race is based on robust evidence and statistical analysis; in this case, rationale for inclusion of race is based on extensive data demonstrating increased rates of preeclampsia in Black patients [5, 6, 13] s, we should consider if there is a plausible causal mechanism for the racial difference that justifies the race correction. Both the Ecosocial Theory of Disease Distribution and Fundamental Cause Theory help explain this increased risk of preeclampsia in patients identifying as Black; increased burden of disease stems from the sequelae of current and historical racism at all levels, and is influenced by access to care, environmental exposures, and social determinants of health [5, 6, 38]. Third, providers should consider whether implementing a race correction will relieve or exacerbate the health inequity. While limited data exists on this topic, the inclusion of Black race as a risk factor should expand access to a therapy expected to relieve, rather than exacerbate, the disparity.

Quality improvement has been suggested as a way to standardize healthcare and promote health equity, but care is needed to assure quality improvement is equity-focused and does not inadvertently widen disparities [27, 37]. Research studies should be conducted to determine whether the prescription of ASA to Black patients consistently decreases rates of preeclampsia and associated morbidity and mortality. Does this apply to all Black women? Is the effect more pronounced in US-born Black women, foreign-born Black women, or is it similar? This evaluation is crucial, as ineffective therapes can be wasteful, harmful, and exacerbate mistrust.

Strengths of the intervention include implementation of a sustainable system which should not increase provider visit time and is scalable to large provider groups. Similarly, the built-in checklist can be updated on a systems-wide level when adjustments to the USPSTF screening are published, reducing individual error or lag in adopting screening measures. Another vital strength of this intervention is the equitable prescription of ASA, and built-in mechanism to decrease implicit bias. Our standardized approach resulted in similar rates of correct prescriptions for ASA in Black and non-Black patients, suggesting a mechanism for delivering equitable preventative obstetric care. Our standardized screening structure can also be adapted to other disease processes with published screening guidelines.

Limitations of this project exist. This quality improvement initiative focused on screening and prescription of ASA to patients at moderate to high risk of preeclampsia, and did not evaluate downstream effects of preeclampsia and associated sequelae. As this project was conducted at a single institution, the results may not be generalizable in different patient populations. Future research efforts should evaluate whether our success with standardizing the ASA screening process can be replicated at other institutions. A cost analysis of the intervention could be beneficial, however the authors anticipate this to be a cost-effective intervention given the shift from provider-based screening time towards RN-based screening time. Additionally, further studies should evaluate downstream effects of standardized screening and recommendation of ASA, including rates of compliance with ASA and rates of development of preeclampsia after implementation of standardized screening and prescription.

## Conclusions

Our intervention resulted in a feasible and sustainable way to standardize screening and implementation of ASA to patients at elevated risk for preeclampsia. We observed that this protocol created a reproducible way to ensure consistency with screening and recommending ASA throughout a large practice with multiple providers. Standardizing the screening process decreases the risk of subconscious biases impacting any specific provider’s tendency to prescribe ASA to applicable patients. Providers at other institutions may find it easy to reproduce or adapt our QI processes to improve delivery of reliable preventative obstetric care.

At our institution, a strikingly high percentage of Black patients warrant a recommendation for ASA to reduce preeclampsia risk, as per the 2021 USPSTF guidelines. Our standardized approach to prenatal screening and prescription of ASA resulted in similar rates of correct prescriptions for ASA in Black and non-Black patients, suggesting this intervention was a mechanism for delivering equitable preventative obstetric care.

## Data Availability

The datasets used and/or analyzed during the current study are available from the corresponding author on reasonable request, with deidentification of data.
